# The effect of hypoxia-inducible factor 1-alpha on hypoxia-induced apoptosis in primary neonatal rat ventricular myocytes

**Published:** 2010-02

**Authors:** Yan-Fang Zhou, Xiao-Wei Zheng, Guo-Xian Qi, Guo-Hui Zhang, Zhi-Hong Zong

**Affiliations:** Department of Cardiology, First Affiliated Hospital of China Medical University, Shenyang, China; Cardiovascular Disease Institute, First People’s Hospital, Zhenjiang, China; Department of Cardiology, First Affiliated Hospital of China Medical University, Shenyang, China; Department of Cardiology, First Affiliated Hospital of China Medical University, Shenyang, China; Cardiovascular Disease Institute, First People’s Hospital, Zhenjiang, China; Department of Biochemistry, China Medical University, ShenYang, China

**Keywords:** HIF-1, primary neonatal rat ventricular myocytes, hypoxia, apoptosis

## Abstract

**Aim:**

To study the role of hypoxia-inducible factor 1-alpha (HIF-1α) on hypoxia-induced apoptosis in primary neonatal rat ventricular myocytes.

**Methods:**

Primary neonatal rat ventricular myocytes were exposed to hypoxia for 24 hours. HIF-1α activity was suppressed by treating the cells with 3-(5′-hydroxymethyl-2′-furyl)-1-benzyl indazole (YC-1). The degree of cell apoptosis was assessed by Hoechst 33258 DNA staining. The levels of HIF-1α and the pro-apoptotic proteins Bnip3, Bax and Bad were measured with western blotting.

**Results:**

On exposure to hypoxia, there was an increase in the expression levels of HIF-1α, and the pro-apoptotic protein Bnip3 was upregulated. Suppression of HIF-1α activity by YC-1 treatment was followed by blockade of hypoxia-induced apoptosis and Bnip3 expression; however, the changes in the levels of Bax and Bad expression were unclear.

**Conclusion:**

Acute hypoxia enhanced primary neonatal rat ventricular myocyte apoptosis through the activation of HIF-1α and a mechanism that perhaps involved Bnip3. Targeting HIF-1α may be a new strategy for reducing the degree of hypoxia-induced apoptosis in ventricular myocytes.

## Summary

Currently, it is believed that cell death in ventricular myocytes occurs via not only necrosis but also apoptosis. Necrosis is a destructive and uncontrolled process, whereas apoptosis is a highly regulated process of programmed cell suicide. The highly organised nature of apoptotic signalling renders it amenable to manipulation. However, the exact regulating mechanisms of apoptosis in ventricular myocytes remain unclear. A better understanding of the apoptotic pathways and their regulatory mechanisms might help identify novel therapeutic targets for alleviating myocardial damage and dysfunction.

HIF-1 is a transcription factor composed of a strictly regulated α-subunit and a constitutive β-subunit.^1^,^2^ HIF-1α is mainly regulated by hypoxia; this protein undergoes rapid ubiquitination and proteasomal degradation in the normal condition, but under hypoxic conditions, the degradation of HIF-1α is suppressed and it is translocated to the nucleus, where it dimerises with HIF-1β.^3^ The heterodimeric HIF-1 molecule binds to hypoxia-responsive elements (HRE) located in the promoter and enhancer regions of hypoxia-regulated genes, causing their transactivation. HIF-1α is thought to be a crucial regulator of hypoxia-adaptational responses.^4^-^6^

Some genes that encode important proteins involved in the apoptotic pathway, such as some of the Bcl-2 family proteins, are regulated by HIF-1α. It was reported that the pro-apoptotic proteins Bnip3, Bad and Bax^7^-^9^ are regulated by HIF-1α. HIF-1α exerts both anti-apoptotic and pro-apoptotic effects, depending on the cell type.^10^ This differential effect is partly due to the regulatory effect of HIF-1α on the pro-apoptotic proteins on the Bcl-2 family.

To understand the function of HIF-1α in hypoxia-induced apoptosis in primary neonatal rat ventricular myocytes and the underlying molecular mechanisms, we induced apoptosis in primary neonatal rat ventricular myocytes by subjecting them to hypoxic conditions for 24 hours. HIF-1α activity was suppressed by treating the cells with YC-1, which is an HIF-1α inhibitor used widely in both *in vitro* and *in vivo* studies.^11^ We observed that hypoxia increased the expression levels of HIF-1α and proapoptotic protein Bnip3 and the degree of apoptosis; however, when HIF-1α was inhibited by YC-1, there was a corresponding decrease in the level of Bnip3 protein expression and the degree of apoptosis.

## Methods

## Cell culture, hypoxia, and inhibition of HIF-1α

The hearts of one- to two-day-old rats were rapidly removed from the chest cavity under anesthesia; the heart samples were trimmed of atrial tissue, great vessels and pericardium. The ventricles were washed in ice-cold phosphate-buffered saline (PBS), cut into small pieces of about 1 mm^3^ and digested with 0.1% trypsin (Sigma). Cells were harvested after digestion and resuspended in Dulbecco’s modified Eagle medium (DMEM)/F12 (1:1) (GIBCO) supplemented with 10% (v/v) foetal bovine serum (FBS, Hyclone), 100 U/ml penicillin and 100 μg/ml streptomycin. The fibroblast content in the cell suspension was reduced using a differential attachment method.

The cell suspension was transferred to 100-mm plastic culture dishes (Corning), which were placed in an incubator at 37°C for 90 min. The myocytes remaining in suspension were then plated onto new plastic culture dishes at a density of 5 × 10^5^ cells/ml for culturing. Cell viability at plating was assessed by trypan blue exclusion. To test the purity of the myocytes, they were subjected to immunocytochemical staining for expression of myocardial sarcomeric actin. On the fourth day of culturing, the cells were classified into various groups and incubated under normoxic (20% O_2_) or hypoxic (5% O_2_, 2% O_2_, 1% O_2_) conditions. In addition, cells cultured under conditions of 1% O_2_ were given 5 μmol/l YC-1 to inhibit HIF-1α activity.

## Hoechst 33258 DNA staining

Nuclear staining with Hoechst 33258 was assessed to detect chromatin condensation or nuclear fragmentation, which are characteristic of apoptosis. Cells cultured on glass slides were fixed with 4% paraformaldehyde and stained with 1 μg/ml Hoechst 33258 (Sigma) for 10 min at room temperature. The cells were then washed three times with sterilised, distilled H_2_O. Cells were counted and 200 were isolated and scored for the incidence of apoptotic chromatin changes using a fluorescence microscope (TE 300, Nikon). Three independent investigators counted the cells.

## Protein extraction and western blotting

Cells were washed and scraped from the dishes. Cellular total protein was extracted by five packed-cell volumes of ice-cold lysis buffer (containing 10 mM Tris-HCl, pH 7.8; 1.5 mM ethylenediamine tetra-acetic acid (EDTA); 10 mM KCl; 0.5 mM dithiothreitol (DTT); 1 mM sodium orthovanadate; 2 mM levamisole; 0.5 mM benzamidine; and 0.05% Nonidet P-40) containing a protease inhibitor cocktail (Sigma), and three rounds of sonication (5 s, 4°C). Protein concentrations were determined using the Bio-Rad Bradford assay kit (Bio-Rad). Equal amounts of total proteins were separated by sodium dodecyl sulfate-polyacrylamide gel electrophoresis (SDS-PAGE) and then transferred to Immobilon-P membranes (Millipore). Membranes were blocked with 5% non-fat milk at room temperature for one hour and then incubated overnight at 4°C with primary antibodies, then incubated using a secondary antibody and detected using the diaminobenzidine detection kit (DAB kit, Amersham Pharmacia).

The primary antibodies used were antibodies to HIF-1α (H-206, Santa Cruz), Bax (N20, Santa Cruz), Bad (C20, Santa Cruz), Nip3 (C-18, Santa Cruz), and actin (Act40, Sigma). The secondary antibody was donkey anti-goat IgG-HRP or goat anti-rabbit IgG-HRP (Santa Cruz). For quantification purposes, densitometric measurements were performed using the Quantity One 1-D analysis software for Windows (Bio-Rad). The data from the western blot analysis were expressed as relative density/β-actin.

## Data analysis

The results were expressed as mean ± standard deviation (SD). For multiple comparisons, results were analysed by analysis of variance (ANOVA) and the least-significant difference *post-hoc* test was used to identify significant differences between the individual cell groups; *p* < 0.05 was considered as statistically significant. All statistical analyses were performed using the SPSS 11.5 software.

## Results

## Effect of hypoxia

**Fig. 1.**
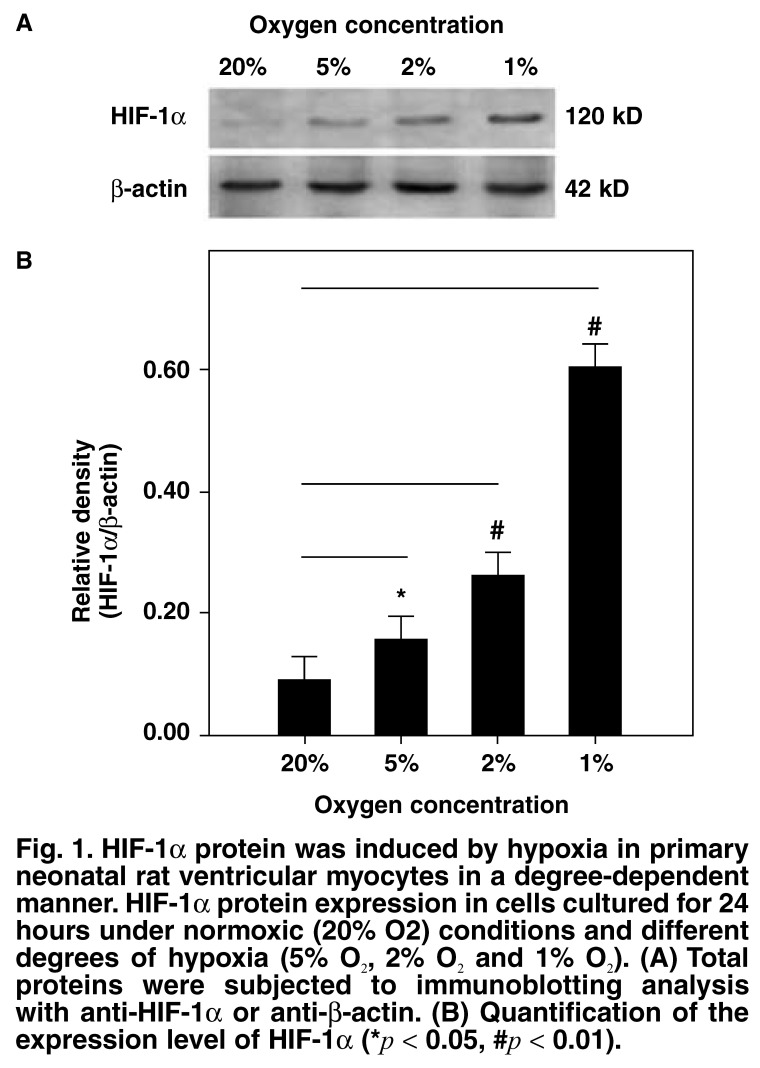
HIF-1α protein was induced by hypoxia in primary neonatal rat ventricular myocytes in a degree-dependent manner. HIF-1α protein expression in cells cultured for 24 hours under normoxic (20% O_2_) conditions and different degrees of hypoxia (5% O_2_, 2% O_2_ and 1% O_2_). (A) Total proteins were subjected to immunoblotting analysis with anti-HIF-1α or anti-β-actin. (B) Quantification of the expression level of HIF-1α (*p < 0.05, #*p* < 0.01).

First, we investigated whether exposure to hypoxia would increase the expression level of HIF-1α and the degree of apoptosis in primary neonatal rat ventricular myocytes. Primary neonatal rat ventricular myocytes on the fourth day of culture were incubated under conditions of normoxia or different degrees of hypoxia. Our data showed that under the normoxic condition, the level of HIF-1α expression was low. As expected, the expression level of HIF-1α increased significantly (*p* < 0.05, *p* < 0.01; Fig. 1) in response to hypoxia in a manner dependent on the degree of hypoxia.

**Fig. 2.**
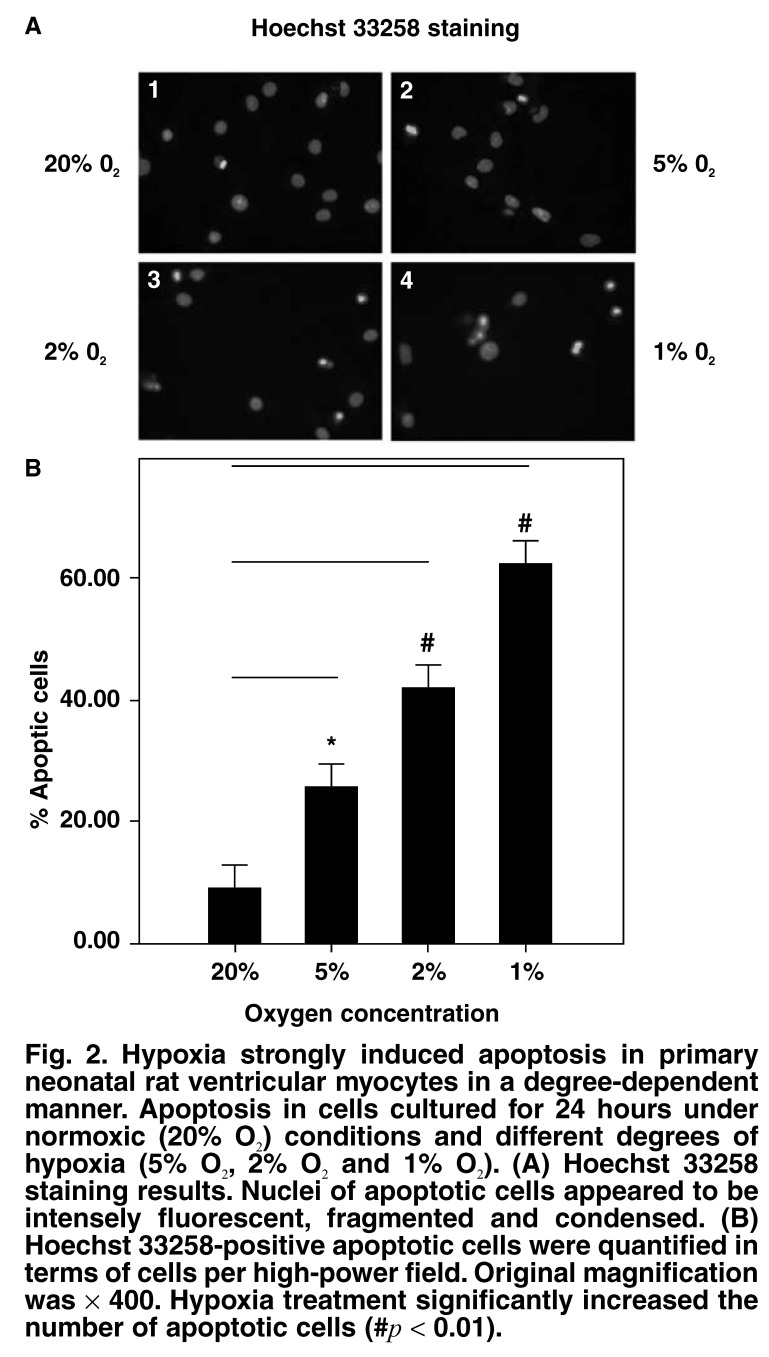
Hypoxia strongly induced apoptosis in primary neonatal rat ventricular myocytes in a degree-dependent manner. Apoptosis in cells cultured for 24 hours under normoxic (20% O_2_) conditions and different degrees of hypoxia (5% O_2_, 2% O_2_ and 1% O_2_). (A) Hoechst 33258 staining results. Nuclei of apoptotic cells appeared to be intensely fluorescent, fragmented and condensed. (B) Hoechst 33258-positive apoptotic cells were quantified in terms of cells per high-power field. Original magnification was × 400. Hypoxia treatment significantly increased the number of apoptotic cells (#*p* < 0.01).

The apoptotic rate in the ventricular myocytes cultured under hypoxic conditions was significantly higher than that in the controls; the increase in the apoptotic rate of the former increased with the degree of hypoxia (apoptotic rate: 9 ± 2% in cells cultured under normoxic conditions; 26 ± 5.4% in cells cultured at 5% O_2_; 42 ± 6.2% in cells cultured at 2% O_2_; and 62 ± 5.4% in cells cultured at 1% O_2_ (*p* < 0.01; Fig. 2).

These data showed that HIF-1α seemed to be partly responsible for the apoptosis in primary neonatal rat ventricular myocytes cultured under hypoxic conditions.

## Effect of HIF-1α inhibitor

**Fig. 3.**
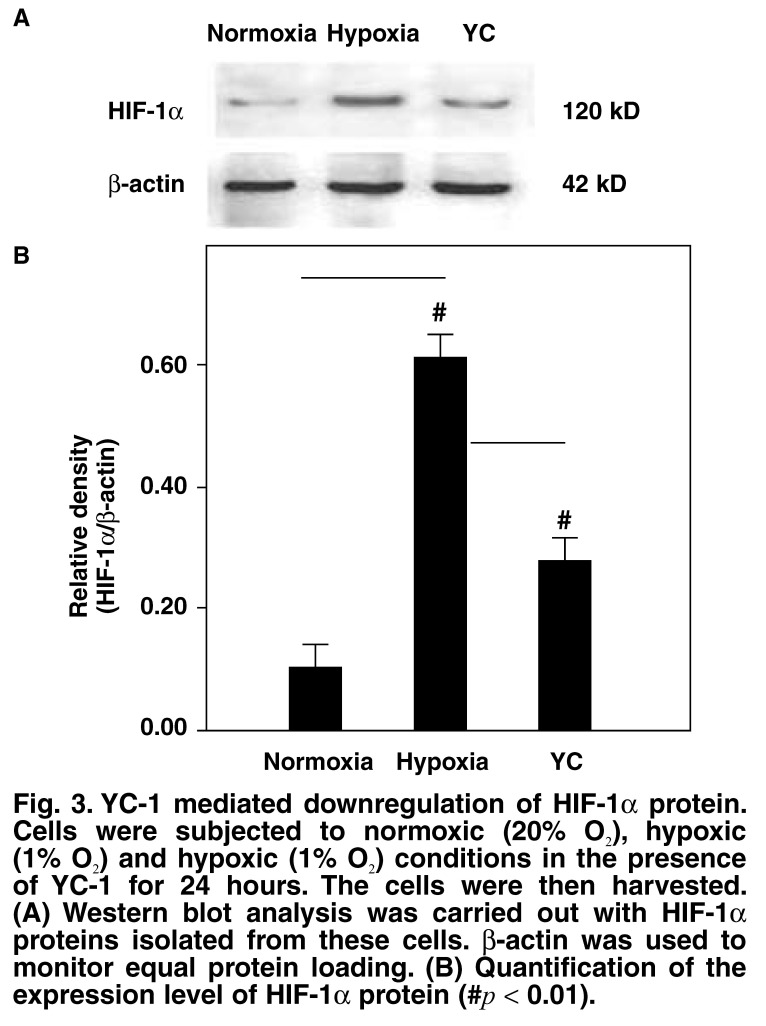
YC-1 mediated downregulation of HIF-1α protein. Cells were subjected to normoxic (20% O_2_), hypoxic (1% O_2_) and hypoxic (1% O_2_) conditions in the presence of YC-1 for 24 hours. The cells were then harvested. (A) Western blot analysis was carried out with HIF-1α proteins isolated from these cells. β-actin was used to monitor equal protein loading. (B) Quantification of the expression level of HIF-1α protein (#*p* < 0.01).

To confirm that the apoptotic response to hypoxia in primary neonatal rat ventricular myocytes was mediated specifically by HIF-1α, the cells were treated with YC-1, a small-molecule inhibitor of HIF-1α. Cells were cultured at 1% O_2_ in the presence of YC-1 (5 μmol/l) for 24 hours and were analysed by immunoblotting for HIF-1α expression. Under hypoxic condition of 1% O_2_, HIF-1α activity in cells cultured in the presence of YC-1 was significantly less than that in those cultured in the absence of YC-1 (*p* < 0.01; Fig. 3).

**Fig. 4.**
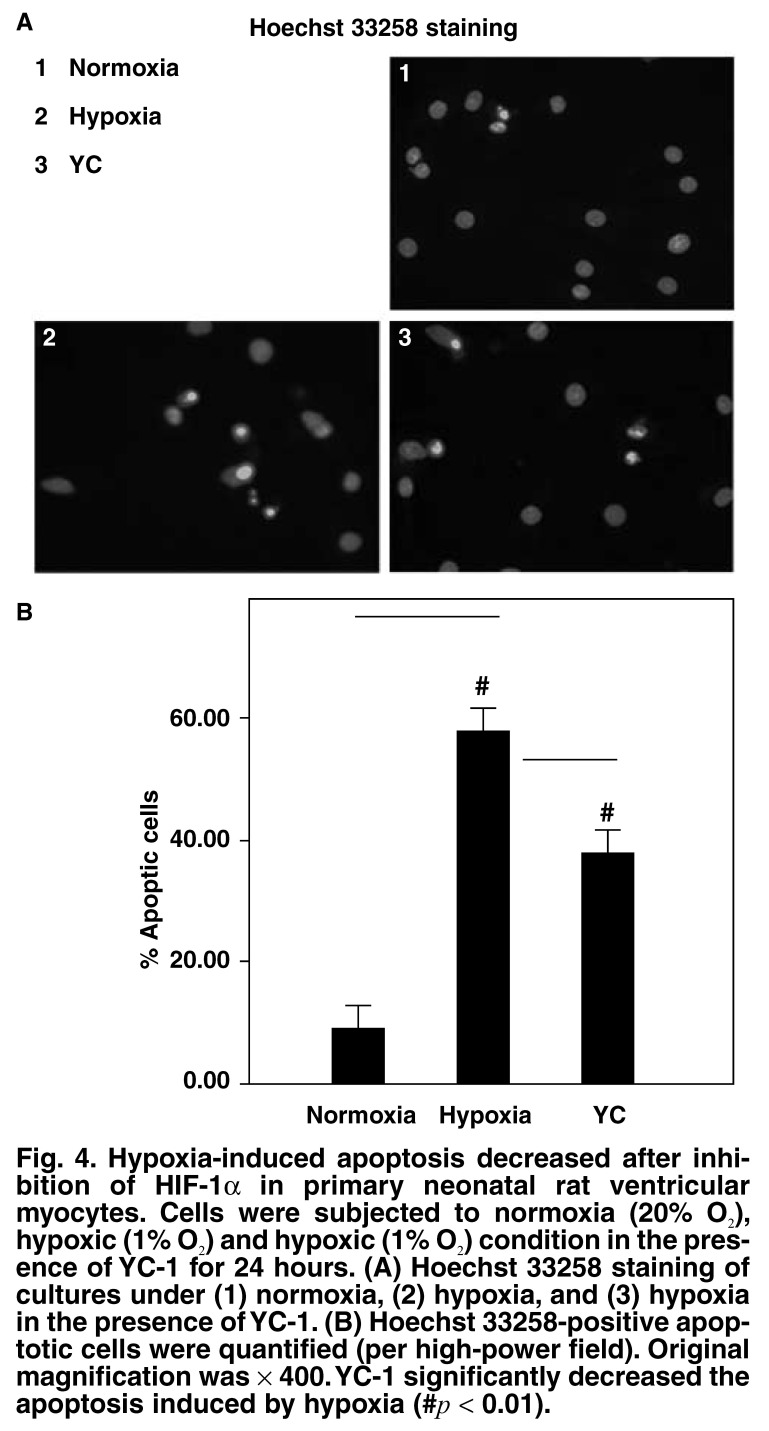
Hypoxia-induced apoptosis decreased after inhibition of HIF-1α in primary neonatal rat ventricular myocytes. Cells were subjected to normoxia (20% O_2_), hypoxic (1% O_2_) and hypoxic (1% O_2_) condition in the presence of YC-1 for 24 hours. (A) Hoechst 33258 staining of cultures under (1) normoxia, (2) hypoxia, and (3) hypoxia in the presence of YC-1. (B) Hoechst 33258-positive apoptotic cells were quantified (per high-power field). Original magnification was × 400. YC-1 significantly decreased the apoptosis induced by hypoxia (#*p* < 0.01).

On selective suppression of HIF-1α activity by YC-1, there was a decrease in the degree of hypoxia-induced apoptosis. Under hypoxic conditions, the rate of apoptosis in cells cultured in the presence of YC-1 was nearly 20% less than that in cells treated in the absence of YC-1 (58 ± 7.9% and 38 ± 2.6% in cells cultured in the absence and presence of YC-1, respectively) (*p* < 0.01; Fig. 4). These findings confirmed that a decrease in the rate of apoptosis was specifically due to a reduction in the level of HIF-1α.

## Effects of hypoxia and HIF-1α inhibitor on pro-apoptotic proteins

**Fig. 5.**
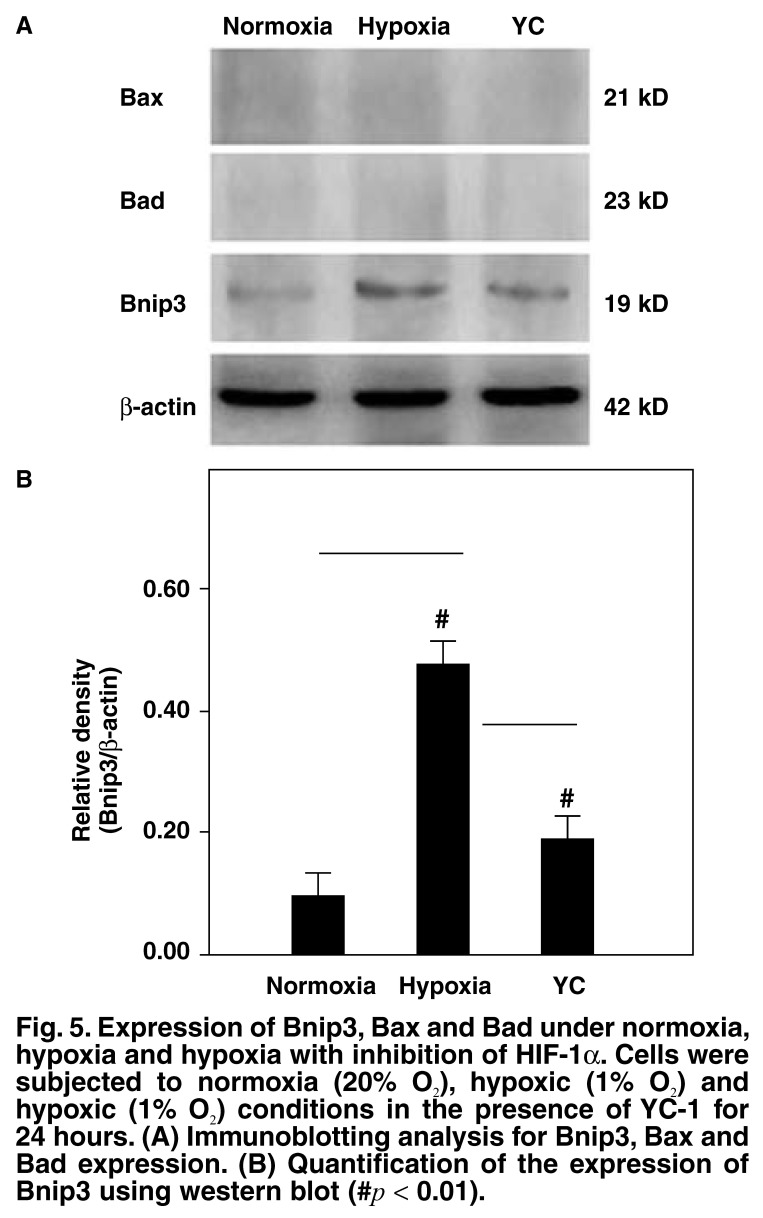
Expression of Bnip3, Bax and Bad under normoxia, hypoxia and hypoxia with inhibition of HIF-1α. Cells were subjected to normoxia (20% O_2_), hypoxic (1% O_2_) and hypoxic (1% O_2_) conditions in the presence of YC-1 for 24 hours. (A) Immunoblotting analysis for Bnip3, Bax and Bad expression. (B) Quantification of the expression of Bnip3 using western blot (#*p* < 0.01).

To further investigate the underlying mechanisms by which HIF-1α enhances the hypoxia-induced apoptosis in primary neonatal rat ventricular myocytes, we evaluated the expression levels of the pro-apoptotic proteins Bnip3, Bax and Bad. The Bnip3 expression level when the cells were cultured at 1% O_2_ was significantly higher than that under normoxic conditions (*p* < 0.05; Fig. 5). When HIF-1α activity was inhibited by treating the cells with YC-1 and there was a significant reduction in the apoptotic rate of cells compared to that under hypoxic conditions without YC-1, there was a simultaneous reduction in the expression level of Bnip3 (*p* < 0.05; Fig. 5). No clear-cut trends in the expression levels of the other pro-apoptotic proteins Bax and Bad were identified. These results suggest that HIF-1α probably mediated the occurrence of hypoxia-induced apoptosis by enhancing the activation of Bnip3.

## Discussion

In many cell types, HIF-1α is rapidly degraded and cannot be detected under normoxic conditions. Hypoxia can induce HIF-1α protein accumulation and mediate a hypoxia-adaptational response. Hypoxia is a characteristic feature of cardiac ischaemia. We cultured primary neonatal rat ventricular myocytes under different levels of hypoxia and examination of these cells revealed that the protein levels of HIF-1α in rat ventricular myocytes increased significantly under hypoxic conditions, depending on the degree of hypoxia (Fig. 1). In addition, as with others culturing cell types, our data showed that there was low accumulation of HIF-1α under normoxic conditions in primary neonatal rat ventricular myocytes (Fig. 1). This accumulation may be associated with the growth factor present in the medium and serum. Previous studies have suggested that some growth factors could stimulate expression of HIF-1α under normoxic conditions.^12^-^15^

Some studies have indicated that HIF-1 plays both an antiapoptotic and pro-apoptotic role, depending on the cell type. In our experiment, Hoechst 33258 DNA staining revealed that hypoxia could induce apoptosis in primary neonatal rat ventricular myocytes and that the degree of apoptosis depended on the degree of hypoxia (Fig. 2). These findings suggested that there might be an association between hypoxia-induced apoptosis and the accumulation of HIF-1α.

To elucidate the effect of this factor on hypoxia-mediated apoptosis in primary neonatal rat ventricular myocytes, we cultured these cells in the presence of YC-1, an inhibitor of HIF-1α. YC-1 blocked the expression of HIF-1α, which was induced by hypoxia, iron chelation and proteasomal inhibition, and also degraded ectopically expressed HIF-1α.^11^ YC-1 inhibits HIF-1α activity *in vitro*, and *in vivo* and has no serious toxic effects;^16^ 5 μmol/l YC-1 could have resulted in approximate reduction of HIF-1α proteins compared with short hairpin RNAs (hrRNAs) against HIF-1α.^17^ Therefore, in our experiment, instead of genetic manipulation, we chose YC-1 for the inhibition of HIF-1α. It has been shown that, when YC-1 was added to hypoxia-treated cells, the level of HIF-1α expression decreased (Fig. 3), with a simultaneous decrease in the apoptotic rate of cells (Fig. 4). These data demonstrated that HIF-1α mediated apoptosis induced by hypoxia in cultured primary neonatal rat ventricular myocytes.

Some reports have described a possible role of HIF-1α in the modulation of apoptosis by inducing the transcription of different Bcl-2 pro-apoptotic members and other proteins involved in apoptosis. Bnip3 is a member of the Bcl-2 family proteins, which display pro-apoptotic activity. This protein contains the Bcl-2 homology (BH3) and a single carboxy-terminal membrane-anchoring domain (TM), which targets specific intracellular organelles, especially mitochondria.

Recent studies have shown that BNIP3 undergoes a dual subcellular localisation and initiates different cell death-signalling processes in the endoplasmic reticulum and mitochondria in a rat dopaminergic neuronal cell line.^18^ Bnip3 promoter was identified to contain the binding sites for HIF-1α.^9^,^19^ Elevated levels of Bnip3 expression have been observed *in vivo* in an animal model of chronic heart failure^20^ and have been found to be expressed in considerable amounts in the adult myocardium.^21^ Moreover, Bnip3 expression has been reported to be upregulated in neonatal rat ventricular myocytes subjected to hypoxia, resulting in mitochondrial dysfunction and subsequent cell death.^9^,^22^ Bnip3 has been demonstrated to induce both necrotic and apoptotic cell death.^22^-^24^ However, investigations must be carried out to determine whether HIF-1α or alternative cellular factors mechanistically account for the induction of Bnip3 during hypoxic injury of ventricular myocytes.

With western blot analysis, we observed an increase in the expression level of Bnip3 in cells exposed to hypoxic conditions. When YC-1 was added to cells exposed to hypoxic conditions, there was a decrease in the expression level of HIF-1α and a simultaneous decrease in the that of Bnip3. The concurrent changes in the expression levels of two pro-apoptotic proteins of the Bcl-2 family, Bax and Bad were unclear.

Due to a shortage of funds, we have not further tested the role of Bnip3 expression in HIF-1α-mediated apoptosis in cultured primary neonatal rat ventricular myocytes. The present results could suggest a possible link between hypoxia, HIF-1α signalling and the activation of genes encoding Bnip3, which are involved in apoptosis in primary neonatal rat ventricular myocytes.

## Conclusions

We found that exposure to hypoxic conditions for 24 hours increased the levels of HIF-1α expression in a manner that was dependent on the degree of hypoxia. The apoptotic rate in ventricular myocytes was also increased in hypoxia-exposed cells in a manner dependent on the degree of hypoxia. The expression level of the pro-apoptotic protein Bnip3 was upregulated under hypoxic conditions. When HIF-1α activity was suppressed by treatment with YC-1, the hypoxia-induced apoptosis and Bnip3 expression were blocked.

We concluded that HIF-1α mediated apoptosis in primary neonatal rat ventricular myocytes cultured under acute hypoxic conditions, and that the pro-apoptotic protein Bnip3 may be one of the key molecules involved in this effect of HIF-1α. Targeting HIF-1α may represent a new strategy for reducing ventricular myocyte apoptosis induced by hypoxia.

## References

[1] Wang GL, Semenza GL (1995). Purification and characterization of hypoxiainducible factor 1.. J. Biol Chem.

[2] Wang GL, Jiang BH, Rue EA, Semenza GL (1995). Hypoxia-inducible factor 1 is a basic-helix–loop–helix-PAS heterodimer regulated by cellular O_2_ tension.. Proc Natl Acad Sci USA.

[3] Reyes H, Reisz-Porszasz S, Hankinson O (1992). Identification of the Ah receptor nuclear translocator protein (Arnt) as a component of the DNA binding form of the Ah receptor.. Science.

[4] Semenza GL (2000). Surviving ischemia: adaptive responses mediated by hypoxia-inducible factor 1.. J Clin Invest.

[5] Wenger RH, Stiehl DP, Camenisch G (2005). Integration of oxygen signaling at the consensus HRE.. Sci STKE.

[6] Semenza GL (2006). Development of novel therapeutic strategies that target HIF-1.. Expert Opin Ther Targets.

[7] Bruick RK (2000). Expression of the gene encoding the proapoptotic Nip3 protein is induced by hypoxia.. Proc Natl Acad Sci USA.

[8] Merighi S, Benini A, Mirandola P, Gessi S, Varani K, Leung E (2007). Hypoxia inhibits paclitaxel-induced apoptosis through adenosine-mediated phosphorylation of Bad in glioblastoma cells.. Molec Pharmacol.

[9] Chen JK, Hu LJ, Wang J, Lamborn KR, Kong EL, Deen DF (2005). Hypoxiainduced BAX overexpression and radiation killing of hypoxic glioblastoma cells.. Radiat Res.

[10] Piret JP, Mottet D, Raes M, Michiels C (2002). Is HIF-1alpha a pro- or an antiapoptotic protein?. Biochem Pharmacol.

[11] Kim HL, Yeo EJ, Chun YS, Park JW (2006). A domain responsible for HIF-1alpha degradation by YC-1, a novel anticancer agent.. Int J Oncol.

[12] Görlach A, Diebold I, Schini-Kerth VB, Berchner-Pfannschmidt U, Roth U, Brandes RP (2001). Thrombin activates the hypoxia-inducible factor-1 signaling pathway in vascular smooth muscle cells: Role of the p22(phox)-containing NADPH oxidase.. Circ Res.

[13] Richard DE, Berra E, Pouyssegur J (2000). Nonhypoxic pathway mediates the induction of hypoxia-inducible factor 1alpha in vascular smooth muscle cells.. J Biol Chem.

[14] Fukuda R, Hirota K, Fan F, Jung YD, Ellis LM, Semenza GL (2002). Insulinlike growth factor 1 induces hypoxia-inducible factor 1-mediated vascular endothelial growth factor expression, which is dependent on MAP kinase and phosphatidylinositol 3-kinase signaling in colon cancer cells.. J Biol Chem.

[15] Stiehl DP, Jelkmann W, Wenger RH, Hellwig-Burgel T (2002). Normoxic induction of the hypoxia-inducible factor 1alpha by insulin and interleukin-1beta involves the phosphatidylinositol 3-kinase pathway.. FEBS Lett.

[16] Chun YS, Yeo EJ, Choi E, Teng CM, Bae JM, Kim MS (2001). Inhibitory effect of YC-1 on the hypoxic induction of erythropoietin and vascular endothelial growth factor in Hep3B cells.. Biochem Pharmacol.

[17] Kamat CD, Thorpe JE, Shenoy SS, Antonio C, Green DE, Warnke LA, Ihnat MA (2007). A long-term ‘memory’ of HIF induction in response to chronic mild decreased oxygen after oxygen normalization.. BMC Cardiovasc Disord.

[18] Zhang L, Li L, Liu H, Borowitz JL, Isome GE (2009). BNIP3 mediates cell death by different pathways following localization to endoplasmic reticulum and mitochondrion. FASEB J.

[19] Sowter HM, Ratcliffe PJ, Watson P, Greenberg AH, Harris AL (2001). HIF-1-dependent regulation of hypoxic induction of the cell death factors BNIP3 and NIX in human tumors.. Cancer Res.

[20] Regula KM, Ens K, Kirshenbaum LA (2002). Inducible expression of BNIP3 provokes mitochondrial defects and hypoxia-mediated cell death of ventricular myocytes.. Circ Res.

[21] Hamacher-Brady A, Brady NR, Logue SE, Sayen MR, Jinno M, Kirshenbaum LA (2007). Response to myocardial ischemia/reperfusion injury involves Bnip3 and autophagy.. Cell Death Differen.

[22] Kubasiak LA, Hernandez OM, Bishopric NH, Webster KA (2002). Hypoxia and acidosis activate cardiac myocyte death through the Bcl-2 family protein BNIP3.. Proc Natl Acad Sci USA.

[23] Velde CV, Cizeau J, Dubik D, Alimonti J, Brown T, Israels S (2000). BNIP3 and genetic control of necrosis-like cell death through the mitochondrial permeability transition pore.. Molec Cell Biol.

[24] Graham RM, Frazier DP, Thompson JW, Haliko S, Li H, Wasserlauf BJ (2004). A unique pathway of cardiac myocyte death caused by hypoxia-acidosis.. J Exp Biol.

